# Smartphone addiction and self-esteem among university students: a moderated mediation and latent profile analysis

**DOI:** 10.3389/fpsyt.2026.1865079

**Published:** 2026-09-21

**Authors:** Shiyu Zhang, Xiao Zhang, Siyu Yuan, Tianpeng Yang, Liang Li

**Affiliations:** 1 Physical Education College, Jiangxi Normal University, Nanchang, China; 2 Physical Education College, Shanghai University of Sport, Shanghai, China; 3 Physical Education College, Jiangxi Vocational and Technical College of Information Application, Nanchang, China; 4 Physical Education College, Nanchang Normal University, Nanchang, China; 5 Physical Education College, Jiujiang University, Jiujiang, China

**Keywords:** physical learning engagement, self-esteem, sleep quality, smartphone addiction, university students

## Abstract

**Objective:**

This study examined the relationship between smartphone addiction and self-esteem among university students, with a particular focus on the mediating roles of sleep quality and physical learning engagement and the moderating role of gender. Both variable centered and person centered approaches were employed.

**Methods:**

In total, 1,130 valid questionnaires were analyzed. The measurement tools included the Mobile Phone Addiction Index (MPAI), the Rosenberg Self-Esteem Scale (SES), the Pittsburgh Sleep Quality Index (PSQI), and the Utrecht Work Engagement Scale–Student (UWES-S). Common method bias tests, descriptive analyses, correlation analyses, and gender difference examinations were carried out in SPSS 26.0. A moderated serial mediation model was estimated using the PROCESS macro with bootstrap resampling, and latent profile analysis (LPA) was conducted in Mplus 8.3.

**Results:**

Smartphone addiction was positively associated with sleep quality (*β* = 0.066, 95% CI [0.048,0.084], *p* < 0.001), negatively associated with physical learning engagement (*β*=−0.107, 95% CI [−0.203,−0.010], *p* = 0.031), and positively associated with self-esteem (*β* = 0.072, 95% CI [0.038,0.106], *p* < 0.001). Sleep quality was positively associated with self-esteem (*β* = 0.402, 95% CI [0.248,0.557], *p* < 0.001). Notably, the positive associations involving self-esteem were directionally inconsistent with most prior literature and should be regarded as unexpected and exploratory. The indirect effect through sleep quality was significant for both female students (effect=0.026, 95% CI [0.014,0.042]) and male students (effect=0.018, 95% CI [0.008,0.029]), whereas the indirect effect (effect=0.007, 95% CI [0.002,0.014]) through physical learning engagement and the serial indirect effect (effect=0.002, 95% CI [0.001,0.005]) were significant only among male students. However, none of the indices of moderated mediation was significant. Gender significantly moderated the direct smartphone addiction–self-esteem association (*β*=−0.055, 95% CI [−0.101,−0.010], *p* = 0.017). In addition, LPA identified three distinct subgroups characterized by different combinations of sleep quality and physical learning engagement, and these subgroups differed significantly in smartphone addiction and self-esteem.

**Conclusion:**

The association between smartphone addiction and self-esteem in university students appears to involve multiple pathways, with sleep quality representing a more stable correlate than physical learning engagement. Gender differences may also be relevant to this association. LPA identified three subgroups based on sleep quality and physical learning engagement that differed significantly in smartphone addiction and self-esteem. These findings provide only preliminary and exploratory evidence for a multifactorial understanding of the association between problematic smartphone use and self-esteem, particularly given the cross-sectional design, modest effect sizes, and the counterintuitive direction of some associations.

## 1 Introduction

Driven by rapid advances in mobile internet and the extensive adoption of smart devices, smartphones have become deeply integrated into university students’ daily lives, influencing their learning, social interactions, and leisure pursuits. Recent research estimates that the global prevalence of internet addiction among college populations is about 41.84% (1), while the prevalence of problematic smartphone use ranges from 36.5% to 67%, with an average of 52% (2). In China, young people especially college students represent one of the fastest-growing groups of smartphone users (3). College students frequently rely on smartphones to accomplish academic and everyday tasks (4). Previous studies have shown that smartphone addiction (SA) is closely associated with a range of adverse outcomes, including anxiety, depression, insomnia, elevated stress, academic procrastination, reduced life satisfaction, and impaired subjective well-being (5–8). Related research has further confirmed that, among college students, SA is manifested not only in frequent and uncontrolled use at the behavioral level, but also in the depletion of cognitive resources, disruption of emotion regulation, and reduction in real-world engagement (9), thereby undermining life satisfaction (10).

Self-esteem (SE) refers to an individual’s overall evaluation of their own worth, competence, and significance, and is widely regarded as one of the core indicators of psychological adaptation and personality development. The university period is not only a critical stage for the deepening of identity formation, but also a key developmental phase during which self-concept and self-evaluation gradually become more stable. Higher levels of SE are generally associated with better mental health, academic adjustment, social competence, and more positive behavioral choices, whereas lower SE is often accompanied by greater emotional distress and a higher risk of addictive behaviors. Existing studies have reported a relatively stable relationship between SE and SA: individuals with low SE may be more likely to seek compensatory gratification through online social interaction, entertainment, and information consumption, whereas SA has been linked to less positive self-evaluation, potentially through processes such as upward social comparison, social anxiety, and peer alienation in online environments (11–13).

Among the factors that may help explain the association between SA and mental health, SQ has been identified as one of the most frequently discussed variables. A large body of evidence indicates that SA is associated with greater sleep disturbance, bedtime procrastination, insomnia symptoms, and poorer subjective sleep quality (SQ) (14–16). Further studies have shown that variables such as rumination, fear of missing out, negative affect, depression, stress, and anxiety may function as mediators or as components of serial mediation pathways linking SA to SQ (17–19). In addition, longitudinal studies have confirmed reciprocal or recursive temporal relationships among problematic smartphone use, bedtime procrastination, SQ, and mental health (20). Higher levels of physical activity have been found to significantly reduce the tendency toward SA among college students (21–23). At the same time, physical activity may also decrease the risk of SA by enhancing SE, core self-evaluation, self-control, psychological resilience, and social adaptation (11, 24, 25). A growing body of evidence indicates that sleep is closely related to school experience and engagement-related outcomes. Poor SQ predicts lower school engagement, less favorable school experience, and poorer academic functioning over time (26, 27). More recent empirical research further suggests that SQ is positively associated with learning engagement (28). Particularly relevant to the present study, recent evidence among Chinese college students has shown that poorer SQ is significantly associated with lower physical learning engagement (PLE), indicating that SQ and PLE may function as interrelated processes rather than isolated correlates (29). At the same time, PLE may constitute an important real-world behavioral pathway linking SA to SE. In educational settings, active participation in physical learning reflects students’ vigor, dedication, and behavioral investment, which may contribute to competence development, positive feedback, and positive self-evaluation. Previous studies have found that SA significantly reduces college students’ learning engagement, with physical activity and academic self-efficacy playing mediating roles in this association (30).

In addition, SQ and PLE may be linked through an inherent sequential process. Previous research has shown that physical activity contributes to better SQ, whereas SA may mediate the association between the two (31–33). Other studies have suggested that SA may influence physical activity behavior through bedtime procrastination, or affect sleep-related outcomes through physical activity (34). These findings provide indirect support for examining a possible serial association pattern of “SA—SQ—PLE—SE”. Finally, gender may represent an important boundary condition in this mechanism. Prior studies have indicated that female students may be more sensitive to SA-related sleep disturbances and negative emotional processes, whereas certain behavioral engagement pathways may be more pronounced among males (35, 36). Therefore, incorporating gender into the moderated model may help to more accurately reveal the heterogeneous mechanisms through which SA is associated with college students’ SE.

## 2 Research hypotheses

### 2.1 Smartphone addiction and self esteem

SA refers to a behavioral state in which individuals develop excessive dependence on smartphones and are unable to effectively regulate the duration and frequency of use, continuing or repeatedly engaging in smartphone use despite its negative consequences for their study, work, daily life, or physical and mental health (37, 38). When individuals experience a diminished sense of self-worth, insufficient competence, or frustrated social belonging in real life, they are more likely to seek compensatory gratification through online media. Accordingly, low SE may constitute an important psychological antecedent of problematic smartphone use. Meanwhile, according to Conservation of Resources theory, time, attention, and emotion-regulation capacity are all finite psychological resources. SA continuously depletes these resources and undermines individuals’ sense of achievement, interpersonal interaction, and self-control in real-life contexts (6, 9). Previous findings suggest that problematic smartphone use is closely related to self-evaluation and psychological adjustment. Excessive smartphone use may be linked to reduced real-life competence, self-control, and interpersonal functioning, and may therefore be associated with SE. Based on this, the following hypothesis is proposed:

H1:SA is significantly associated with SE among college students.

### 2.2 The mediating role of sleep quality

SQ may represent an important mechanism linking SA to SE. A growing body of research has shown that excessive smartphone use is associated with a range of sleep-related problems, including delayed bedtime, insomnia symptoms, shorter sleep duration, and poorer overall SQ (39–41). Frequent nighttime smartphone use may prolong sleep onset, increase physiological and cognitive arousal, and interfere with restorative sleep processes, thereby undermining SQ. Poorer SQ, in turn, may be related to lower SE. Although SE is related to mental health, it is conceptually distinct from broader constructs such as depression, anxiety, and psychological distress, as it primarily reflects an individual’s evaluation of self-worth. Prior studies have shown that poor or insufficient sleep is associated with lower SE, and longitudinal evidence further suggests that sleep problems may predict subsequent declines in SE (42–44). In this sense, SQ may serve as a potential pathway through which SA is associated with SE. Accordingly, the following hypothesis is proposed:

**H2:** SQ mediates the relationship between SA and SE among college students.

### 2.3 The mediating role of physical learning engagement

Physical activity is widely regarded as an important protective factor against SA. A growing body of research has shown that physical activity not only directly reduces the level of SA, but also exerts indirect effects through SE (30), core self-evaluation, self-control, psychological capital, social adaptation, and psychological resilience (45, 46). Previous studies have demonstrated that SA significantly reduces college students’ learning engagement, with physical activity playing a mediating role in this association (47). In the educational context, PLE reflects students’ behavioral investment, energy, and active participation in physical education learning. SA may reduce attention to real-world tasks and diminish active participation in physical learning contexts. Lower PLE may, in turn, be associated with fewer opportunities for competence affirmation and positive feedback, and may therefore be related to SE (48). Thus, the following hypothesis is proposed:

**H3:** PLE mediates the relationship between SA and SE among college students.

### 2.4 The serial mediating role of sleep quality and physical learning engagement

High-quality sleep is generally thought to be related to the restoration of physical energy, attentional resources, and behavioral initiative, and may therefore be relevant to learning engagement. In contrast, poor SQ may increase fatigue and reduce classroom attention, memory performance (49), and behavioral engagement (27). This influence may be even more pronounced in physical education classes, which inherently require relatively high levels of energy and active behavioral participation. Existing studies have shown that SA may affect sleep-related outcomes through physical activity and bedtime procrastination, while physical activity and SA may jointly influence SQ. Meanwhile, physical activity and real-world participation contribute to the enhancement of SE, competence, and core self-evaluation (26, 50). Based on these findings, the following hypothesis is proposed:

**H4:** SQ and PLE play a serial mediating role in the relationship between SA and SE among college students.

### 2.5 The moderating role of gender

Gender differences have been widely documented in studies of problematic smartphone use, emotional responses, sleep disturbances, and behavioral engagement. Prior studies suggest that females and males may differ in emotional responses to smartphone overuse and in the behavioral consequences associated with real-world participation (35, 51, 52). In addition, gender may also shape one or more indirect pathways in the proposed model. For example, the effect of SA on SQ, the effect of SQ on PLE, or the downstream association with SE may not be equivalent across male and female students. Therefore, gender may condition not only the direct association between SA and SE, but also the strength of the indirect pathways through SQ and PLE. Therefore, the following hypothesis is further proposed (see **Figure 1**):

**Figure 1:**
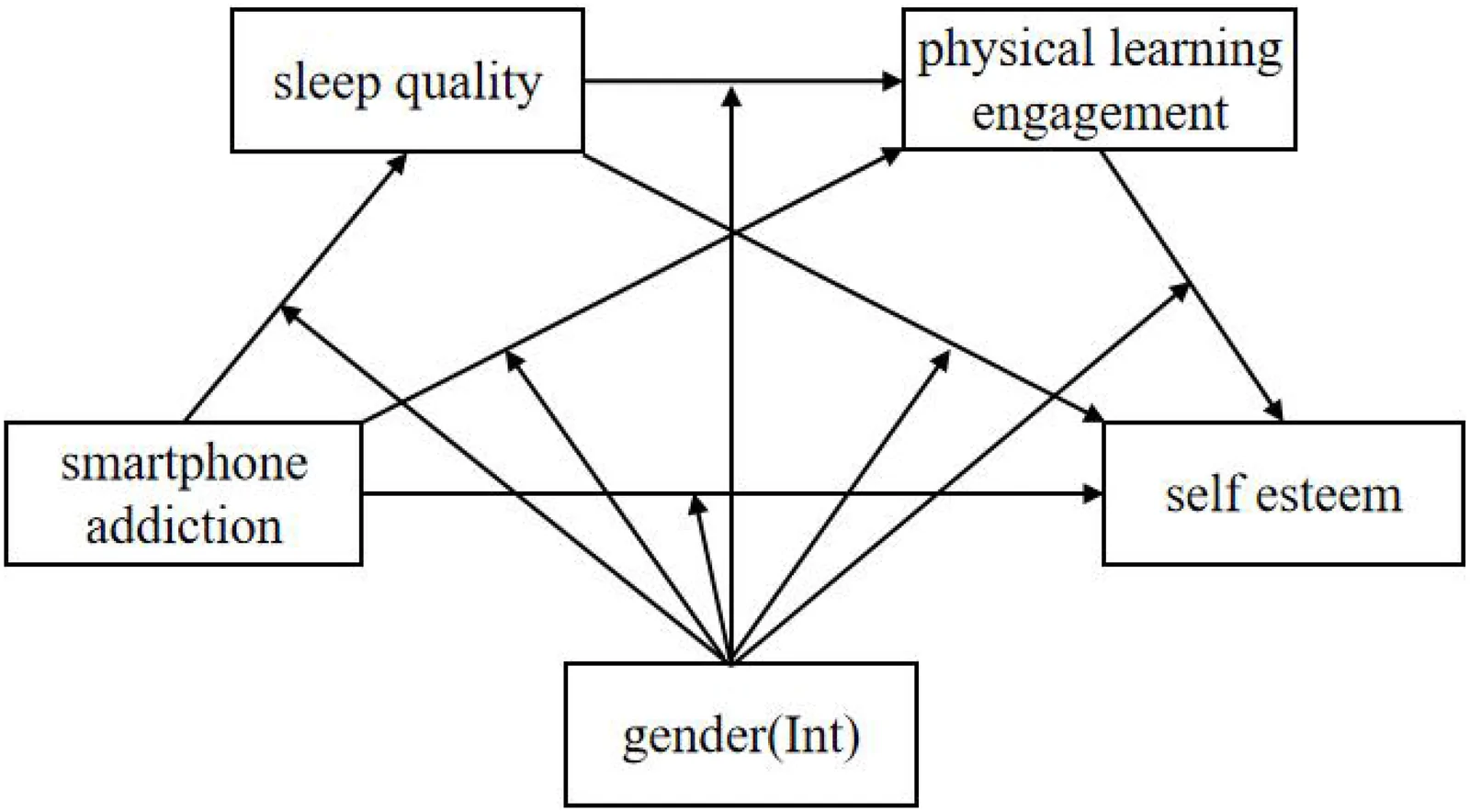
The hypothesized model diagram. Gender(Int)=interaction term between gender and various independent variables (the same below).

H5: Gender moderates the direct association between SA and SE and may also condition the indirect pathways through SQ and PLE.

## 3 Materials and methods

### 3.1 Participants

Sample size was determined using a combination of formal and heuristic considerations. A preliminary lower-bound estimate was obtained in G*Power 3.1 under a simplified linear multiple regression framework, assuming a medium effect size (f^2^ = 0.15), a significance level of α=0.05, and statistical power of 1−*β* = 0.95 (53), the minimum required sample size was estimated to be 119 participants. However, because this calculation was based on a single regression equation, it should be regarded only as a rough reference rather than a formal power analysis for the full moderated serial mediation model. We also considered a commonly used heuristic in psychological and behavioral research suggesting a sample size at least 10 times the number of questionnaire items; with 63 items in total, this yielded a reference value of 630 participants (54). Because the present study involved a more complex analytical framework, including moderated serial mediation, bootstrap estimation of indirect effects, and latent profile analysis, a substantially larger sample was targeted to enhance the stability of parameter estimation and subgroup classification.

Participants were recruited from two universities in Hunan Province, China. To enhance sample heterogeneity, two universities located in Changsha, the provincial capital, were selected as the survey sites. Both universities offer a wide range of academic disciplines and enroll students from relatively diverse backgrounds, which may help capture variation among college students from different academic and social backgrounds. The inclusion criteria for participants were as follows: (1) being a full-time university student; (2) providing informed consent and agreeing to participate voluntarily; and (3) having no diagnosed mental disorders or sleep disorders. The exclusion criteria for questionnaires were: (1) substantial missing data; (2) obviously patterned or repetitive responses; and (3) other clearly invalid responses.

No separate pilot study was conducted. Before the survey was administered, participants were informed of the study purpose, survey content, voluntary nature of participation, and confidentiality assurances. Data collection was conducted only after informed consent had been obtained. Both online and paper-based questionnaires were used. Specifically, 916 online questionnaires and 338 paper-based questionnaires were distributed, accounting for 73.05% and 26.95% of the total, respectively. The average time required to complete the questionnaire was approximately 15 minutes. The formal survey was initiated on January 13, 2026. Based on the study design and sample size estimation principles, 1,254 questionnaires were distributed. After invalid questionnaires were excluded according to the predefined criteria, 1,130 usable questionnaires were retained for the final analyses, representing 90.11% of all distributed questionnaires. Because the exact size of the full eligible student population was not available, a population-based response rate could not be calculated precisely. Participants were aged 18 to 23 (19.67 ± 1.15) years. Among them, male students accounted for 53.81% of the sample, whereas female students comprised 46.19%.

### 3.2 Measurement

#### 3.2.1 Smartphone addiction

SA was assessed using the Mobile Phone Addiction Index (MPAI), originally developed by Leung (55). The scale consists of 17 items (e.g., “I have tried to spend less time on my mobile phone, but I could not”), primarily assessing four dimensions of problematic mobile phone use: inability to control craving, withdrawal/escape, and inefficiency-related problems. The MPAI has demonstrated good applicability and psychometric performance among Chinese college students, with a reported Cronbach’s α of 0.870 (56). Each item was rated on a 5-point Likert scale, with higher scores indicating greater severity of SA.

#### 3.2.2 Self esteem

SE was measured using the Rosenberg Self-Esteem Scale (SES), a widely used instrument developed by Rosenberg to assess individuals’ global sense of self-worth and self-acceptance (57). The scale includes 10 items (e.g., “I hold a positive attitude towards myself”). It is a brief and easy-to-administer self-report measure, with a Cronbach’s α of 0.780 (58). Each item was rated on a 4-point scale, with higher scores indicating higher SE.

#### 3.2.3 Sleep quality

SQ was measured using the Pittsburgh Sleep Quality Index (PSQI), an internationally widely used self-report instrument designed to assess individuals’ overall SQ over the previous month (59). The PSQI contains 19 self-rated items (e.g., “During the past month, have you often felt sleepy?”) and covers seven components, with a reported Cronbach’s α of 0.734 (60). Each item was rated on a 4-point scale, with higher scores indicating poorer SQ.

#### 3.2.4 Physical learning engagement

PLE was assessed using the Utrecht Work Engagement Scale–Student (UWES-S), developed by Schaufeli et al. (61). The scale conceptualizes college students’ PLE across three dimensions: vigor, dedication, and absorption. It contains 17 items (e.g., “I feel full of energy during physical education class”), with a Cronbach’s α of 0.913 (62). All rated on a 5-point Likert scale, with higher scores indicating higher PLE.

### 3.3 Data analysis

All statistical analyses were conducted using SPSS 26.0, except for the assessment of common method bias, which was performed in Mplus 8.3. Common method bias was tested using a single-factor confirmatory factor analysis in which all measurement items were specified to load onto one common latent factor (63). Descriptive statistics, correlation analyses, and tests of gender differences were then conducted. In addition, multicollinearity diagnostics were examined using variance inflation factor (VIF) and tolerance values.

The hypothesized moderated serial mediation model was tested using Hayes’s PROCESS macro (Model 92). PROCESS was used because the study aimed to estimate conditional direct and indirect effects within a regression-based framework using composite scores for all focal constructs, all of which showed acceptable internal consistency. In this model, SA was entered as the independent variable, SE as the dependent variable, SQ and PLE as sequential mediators, and gender as the moderator. Gender was coded as 1 = male and 0 = female, and age was included as a covariate in all equations. Conditional indirect effects were estimated for three pathways: via SQ, via PLE, and via the serial path through SQ and PLE. Their significance was tested using 5,000 bias-corrected bootstrap resamples, with 95% confidence intervals excluding zero indicating statistical significance. Indices of moderated mediation were also examined to test whether these indirect effects varied by gender. Because the focal constructs were assessed using different instruments and scoring ranges, both unstandardized coefficients (*B*) and standardized coefficients (*β*) are reported to facilitate interpretation and comparison of effect sizes across variables.

In addition, to further examine heterogeneity in college students’ psychological and behavioral characteristics from a person centered perspective, latent profile analysis (LPA) was conducted using Mplus 8.3. The models were estimated using robust maximum likelihood estimation (MLR). Model fit was evaluated using the Akaike Information Criterion (AIC), Bayesian Information Criterion (BIC), and sample-size adjusted Bayesian Information Criterion (aBIC), with lower values indicating better fit. The Lo Mendell Rubin adjusted likelihood ratio test (LMR) and the bootstrap likelihood ratio test (BLRT) were employed to assess whether models with additional profiles provided a statistically significant improvement in fit. Entropy values closer to 1 indicated greater classification accuracy (64). The optimal latent profile solution was determined by jointly considering model fit indices, class proportions, classification clarity, and theoretical interpretability. Finally, differences across latent profiles were examined using one-way analysis of variance (ANOVA) when the assumption of homogeneity of variance was met and Welch’s ANOVA when this assumption was violated. *Post hoc* pairwise comparisons were conducted using the Bonferroni correction for variables with homogeneous variances and the Games–Howell procedure for variables with heterogeneous variances.

## 4 Results

### 4.1 Common method bias test

To examine the potential influence of common method bias, a single-factor confirmatory factor analysis was conducted. The single-factor model showed poor fit to the data (CFI = 0.438, RMSEA = 0.142, SRMR = 0.195), with all indices falling outside commonly accepted cutoff criteria. In addition, Harman’s single-factor test was performed by conducting an unrotated principal component analysis on all questionnaire items (65). Eight factors had eigenvalues greater than 1, and the first factor accounted for 27.16% of the total variance, which was below the conventional threshold of 40%. Taken together, these results provide only preliminary statistical evidence suggesting that common method bias is unlikely to fully account for the observed associations. Before conducting the hierarchical regression analyses, multicollinearity and regression assumptions were further examined. All tolerance values were above 0.10 and all variance inflation factor (VIF) values were below 5.00, indicating that multicollinearity was not a serious concern.

The results of the gender difference test showed significant differences between male and female college students in SA, SQ, and PLE (all *p* < 0.001). Specifically, female students reported higher scores than male students on SA and SQ, whereas male students reported higher PLE scores than female students. No significant gender difference was found in SE (*p* = 0.300) (**Table 1**).

**Table 1:** Gender differences (Mann-Whitney U test).

Variable	Female (n = 522)	Male (n = 608)	Mann-Whitney U	Z	*p*
	M ± SD	M ± SD			
SA	38.49 ± 14.09	33.78 ± 14.61	126753.50	-5.852	<0.001
SQ	4.78 ± 3.11	4.23 ± 3.19	140051.00	-3.426	<0.001
PLE	61.10 ± 13.81	64.85 ± 16.72	129608.00	-5.328	<0.001
SE	24.38 ± 5.56	24.51 ± 5.46	153037.50	-1.037	0.300

SQ, sleep quality; PLE, physical learning engagement; SA, smartphone addiction; SE, self-esteem; the same as below.

### 4.2 Descriptive statistics and correlation analysis

As shown in **Table 2**, PLE, SQ, SA, and SE were all significantly correlated with one another (*p* < 0.01). Specifically, PLE was negatively correlated with the other three variables. SQ was significantly positively correlated with SA and SE, and SA was also positively correlated with SE. However, the positive associations of SQ with SE and of SA with SE were directionally inconsistent with conventional theoretical expectations and with much of the prior literature, and should therefore be interpreted cautiously as unexpected findings.

**Table 2:** Correlation analysis results.

Variable	M	SD	PLE	SQ	SA	SE
PLE	63.12	15.55	1			
SQ	4.48	3.17	-0.142^**^	1		
SA	35.95	14.56	-0.161^**^	0.320^**^	1	
SE	24.45	5.51	-0.167^**^	0.228^**^	0.178^**^	1

**p<0.01.

### 4.3 Regression analyses

As shown in **Table 3** and **Figure 2**, When SQ was entered as the dependent variable, SA, gender, age, and the interaction between SA and gender were included in the model. The overall model was significant [*R^2^* = 0.123, *F* = 41.628, *p* < 0.001], explained 12.3% of the variance in SQ. SA was significantly and positively associated with SQ (*β* = 0.309, *p* < 0.001), indicating that higher SA was associated with poorer SQ. Age was also significantly associated with SQ (*β* = 0.160, *p* < 0.001). Neither the main effect of gender nor its interaction with SA reached statistical significance, indicating that gender did not significantly moderate the association between SA and SQ.

**Table 3:** Regression analyses.

Outcome variable	Predictor variable	*R*	*R* ^2^	*F*	*B*	*β*	BootSE	*t*	*p*	95% CI
SQ	SA	0.359	0.123	41.628	0.066	0.309	0.009	7.130	<0.001	[0.048, 0.084]
	Gender				-0.322	-0.067	0.484	-0.665	0.506	[-1.271, 0.627]
	SA×Gender				0.003	0.014	0.012	0.247	0.805	[-0.021, 0.027]
	Age				0.440	0.160	0.077	5.745	<0.001	[0.289, 0.590]
PLE	SA	0.242	0.059	11.652	-0.107	-0.110	0.049	-2.156	0.031	[-0.203,-0.010]
	SQ				-0.532	-0.119	0.225	-2.369	0.018	[-0.973,-0.091]
	Gender				4.161	0.190	2.548	1.633	0.100	[-0.839, 9.162]
	SA×Gender				-0.021	-0.020	0.066	-0.321	0.749	[-0.152, 0.109]
	SQ×Gender				-0.099	-0.020	0.302	-0.328	0.743	[-0.691, 0.493]
	Age				1.669	0.124	0.397	4.206	<0.001	[0.890, 2.447]
SE	SA	0.304	0.092	14.239	0.072	0.111	0.017	4.160	<0.001	[0.038, 0.106]
	SQ				0.402	0.187	0.079	5.117	<0.001	[0.248, 0.557]
	PLE				-0.032	-0.123	0.017	-1.851	0.064	[-0.065, 0.002]
	Gender				4.712	0.123	1.719	2.740	0.006	[1.338, 8.085]
	SA×Gender				-0.055	-0.146	0.023	-2.384	0.017	[-0.101,-0.010]
	SQ×Gender				-0.144	-0.083	0.106	-1.361	0.174	[-0.351, 0.063]
	PLE×Gender				-0.022	-0.062	0.021	-1.035	0.301	[-0.064, 0.020]
	Age				-0.151	-0.032	0.139	-1.081	0.280	[-0.424, 0.123]

*R*, multiple correlation coefficient; *R²*, coefficient of determination; *F*, overall model test statistic; *B*, unstandardized regression coefficient; *β*, standardized regression coefficient; BootSE, bootstrap standard error; 95%CI, 95%confidence interval.

**Figure 2:**
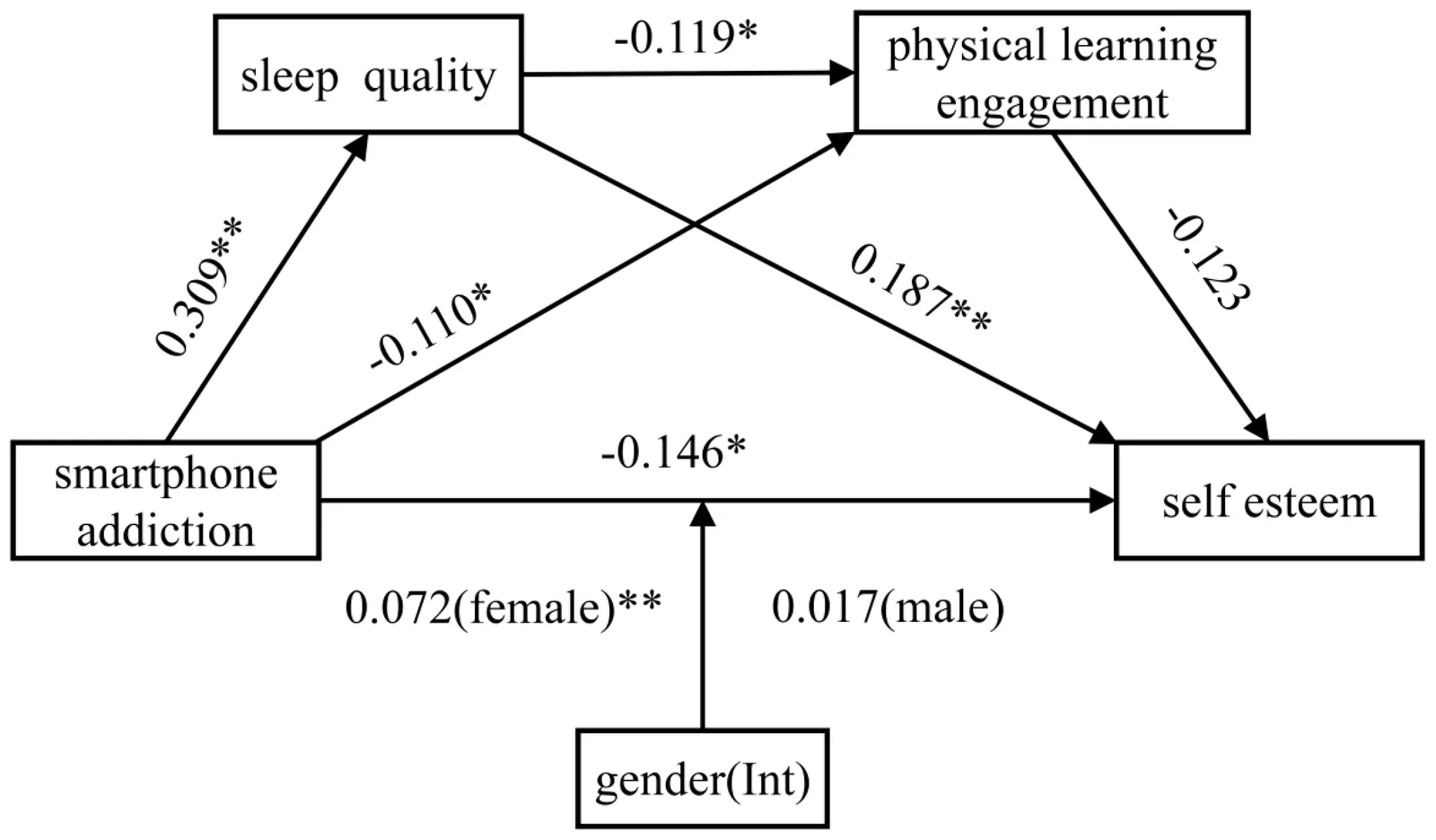
A moderated chain mediation effect model. Excluding paths with insignificant interaction effects (*p*>0.05). *p<0.05; **p<0,001.

When PLE was entered as the dependent variable, SA, SQ, gender, age, the interaction between SA and gender, and the interaction between SQ and gender were included in the model. The model was statistically significant, [*R^2^* = 0.059, *F* = 11.652, *p* < 0.001], although its explanatory power was relatively limited. SA was significantly negatively associated with PLE (*β*=−0.110, *p* = 0.031), indicating that higher levels of SA were linked to lower PLE. SQ was also significantly negatively associated with PLE (*β*=−0.119, *p* = 0.018), suggesting that poorer SQ was related to lower levels of PLE. Age was significantly positively associated with PLE (*β* = 0.124, *p* < 0.001). Neither the main effect of gender (*p* = 0.100) nor any interaction terms were statistically significant, indicating that no significant gender differences were observed in this pathway.

When SE was entered as the dependent variable, SA, SQ, PLE, gender, age, and the interaction terms between gender and the three core predictors were included in the model. The overall model was significant, [*R^2^* = 0.092, *F* = 14.239, *p* < 0.001], explaining 9.2% of the variance in SE. SQ was significantly associated with SE (*β* = 0.187, *p* < 0.001), indicating that poor SQ was associated with higher SE. SA was also significantly associated with SE (*β* = 0.111, *p* < 0.001). This finding deviates from conventional theoretical expectations and may suggest the presence of a suppression effect or greater model complexity, which will be discussed further in the Discussion section. The main effect of gender was significant (*β* = 0.123, *p* = 0.006), indicating a significant gender difference in SE. In addition, the interaction between SA and gender was significantly associated with SE (*β*=−0.146, *p* = 0.017), suggesting that gender moderated the direct association between SA and SE. By contrast, the association between PLE and SE did not reach statistical significance (*β* = −0.123, *p* = 0.064), and neither the SQ × gender (*p* = 0.174) and PLE × gender (*p* = 0.301) interactions nor age showed significant relations with SE.

To improve transparency and assess the robustness of the observed associations, we additionally conducted unadjusted regression analyses with self-esteem as the dependent variable. Consistent with the zero-order correlations, smartphone addiction positively predicted self-esteem in the unadjusted model (B = 0.067, *β* = 0.178, *p* < 0.001, 95% CI[0.045,0.089]), and sleep quality also positively predicted self-esteem (B = 0.396, *β* = 0.228, *p* < 0.001, 95% CI[0.297,0.495]). These associations remained positive in the adjusted models, suggesting that the unexpected direction of the findings was not introduced solely by covariate adjustment.

### 4.4 Mediation analyses

#### 4.4.1 Conditional direct effects

As shown in **Table 4**, after controlling for SQ, PLE, and age, the direct association between SA and SE differed significantly by gender. Specifically, among female college students, the direct effect of SA on SE was significant (Effect=0.072, *p* < 0.001). In contrast, among male college students, this direct effect did not reach statistical significance (Effect=0.017, *p* = 0.288). This finding further supports the moderating role of gender identified in the regression analyses, indicating that the direct pathway from SA to SE was primarily evident among female students.

**Table 4:** Conditional direct effects (SA→ SE, by gender).

Gender	Effect	BootSE	*t*	*p*	LLCI	ULCI
female	0.072	0.017	4.16	<0.001	0.038	0.106
male	0.017	0.016	1.063	0.288	-0.014	0.047

Effect, conditional direct effect of smartphone addiction on self-esteem at each level of gender; BootSE, bootstrap standard error; LLCI, lower limit of the 95% confidence interval; ULCI, upper limit of the 95% confidence interval.

#### 4.4.2 Conditional indirect effects and moderated mediation effects

As shown in **Table 5**, the indirect pathway from SA to SE through SQ was statistically significant among both female students (indirect effect = 0.026, 95% CI [0.014, 0.042]) and male students (indirect effect = 0.018, 95% CI [0.008, 0.029]). However, the corresponding effect sizes were small, and the confidence interval for the index of moderated mediation included zero, indicating that gender did not significantly moderate this indirect pathway.

**Table 5:** Conditional indirect effects and moderated mediation effects.

Path	Gender	Indirect effect	BootSE	BootLLCI	BootULCI
X→M1→Y	female	0.026	0.007	0.014	0.042
	male	0.018	0.006	0.008	0.029
Index of moderated mediation		-0.009	0.009	-0.026	0.008
X→M2→Y	female	0.003	0.003	-0.001	0.010
	male	0.007	0.003	0.002	0.014
Index of moderated mediation		0.004	0.004	-0.005	0.012
X→M1→M2→Y	female	0.001	0.001	-0.000	0.003
	male	0.002	0.001	0.001	0.005
Index of moderated mediation		0.001	0.001	-0.002	0.004

X, smartphone addiction; M1, sleep quality; M2, physical learning engagement; Y, self-esteem; Indirect effect, conditional indirect effect of X on Y through the specified path at each level of gender; BootSE, bootstrap standard error; BootLLCI, lower limit of the bootstrap 95% confidence interval; BootULCI, upper limit of the bootstrap 95% confidence interval.

For the pathway SA→PLE→SE, the indirect effect was not statistically significant among female students, whereas a small indirect effect was observed among male students (indirect effect = 0.007, 95% CI [0.002, 0.014]). Nevertheless, because the confidence interval for the index of moderated mediation included zero, there was no evidence that this indirect pathway significantly differed by gender. Accordingly, this result should be interpreted cautiously and should not be taken as strong evidence of a meaningful gender difference.

For the serial pathway SA→SQ→PLE→SE, the indirect effect was not statistically significant among female students (indirect effect = 0.001, 95% CI [-0.000, 0.003]) and was statistically significant but very small among male students (indirect effect = 0.002, 95% CI [0.001, 0.005]). Again, the confidence interval for the index of moderated mediation included zero, suggesting that gender did not significantly moderate this serial mediation pathway. Given the very small magnitude of these estimates, the serial indirect effect should be regarded as preliminary and of limited practical importance.

Overall, although some indirect effects reached statistical significance, their magnitudes were small. Therefore, these findings should be interpreted as providing limited statistical support rather than strong evidence of substantial practical impact.

#### 4.4.3 Summary of hypothesis testing

As shown in **Table 6**, the proposed hypotheses were partially supported. Specifically, H1 was supported (see **Table 3**), indicating that SA was significantly associated with SE among college students, consistent with the correlation and regression results. H2 was also supported (see **Tables 3**, **5**), suggesting that SQ served as a stable mediator in the relationship between SA and SE, and this indirect effect was generally comparable across genders.

**Table 6:** Summary of hypothesis testing.

Hypothesis	H1	H2	H3	H4	H5
Result	Supported	Supported	Not Supported	Not Supported	Not Supported

By contrast, H3 received only limited support (see **Tables 3**, **5**). Although the mediating effect of PLE was observed among male students, this effect was not consistently significant across gender groups and should therefore be interpreted cautiously as subgroup-specific evidence rather than robust overall support. H4 was not supported (see **Table 5**), indicating that although the serial pathway of SA→SQ→PLE→SE was theoretically plausible, it did not reach statistical significance.

Regarding gender moderation, H5 was not supported (see **Tables 4**, **5**). Although gender significantly moderated the direct association between SA and SE, none of the indices of moderated mediation reached statistical significance. Therefore, the findings support moderation of the direct effect, but not moderated mediation.

### 4.5 Latent profile analysis

As the number of classes increased, the values of AIC, BIC, and aBIC decreased continuously, suggesting progressive improvement in statistical fit. The LMR and BLRT indicated that the two-, three-, four-, and five-class models each fit significantly better than the corresponding model with one fewer class, whereas the six-class model was not supported by the LMR. In terms of classification accuracy, the three-class model yielded the highest entropy value (0.985) among the candidate solutions. In addition, the three classes in the three-class solution accounted for 11.04%, 62.31%, and 26.65% of the sample, respectively, all exceeding the 5% threshold used in the present study to indicate acceptable class size. Although the two-class model was more parsimonious, the three-class model showed better fit, higher classification precision, and identified an additional subgroup that was substantively interpretable. By contrast, the four- and more complex class solutions introduced relatively small classes and appeared to reflect increasing fragmentation rather than clearly distinct and theoretically meaningful profiles. Therefore, According to the recommended guidelines for potential profile analysis (66), considering statistical fit, class size, classification accuracy, parsimony, and theoretical interpretability, the three-class solution was selected as the optimal latent profile structure (see **Table 7**).

**Table 7:** Fit of each potential cross-section model.

Model	AIC	BIC	aBIC	LMR	BLRT	Entropy	Categorical probability%
Class1	74375.744	74617.183	74464.721				
Class2	64304.908	64672.096	64440.228	<0.0001	<0.0001	0.972	30.10%/69.90%
Class3	58059.743	58552.681	58241.405	<0.0001	<0.0001	0.985	11.04%/62.31%/26.65%
Class4	54138.470	54757.157	54366.473	<0.0001	<0.0001	0.981	21.42%/47.52%/7.35%/23.72%
Class5	52922.421	53666.857	53196.766	0.0442	<0.0001	0.982	3.37%/6.85%/20.81%/45.29%/23.67%
Class6	52266.104	53136.289	52586.792	0.1508	<0.0001	0.953	3.35%/6.28%/15.31%/18.67%/33.01%/23.36%

Inspection of the item-response patterns of the three profiles, as illustrated in **Figure 3**, showed that although the three classes displayed generally similar trends across the 24 items, they differed in overall score levels in a relatively stable and stratified manner (see **Figure 3**).

**Figure 3:**
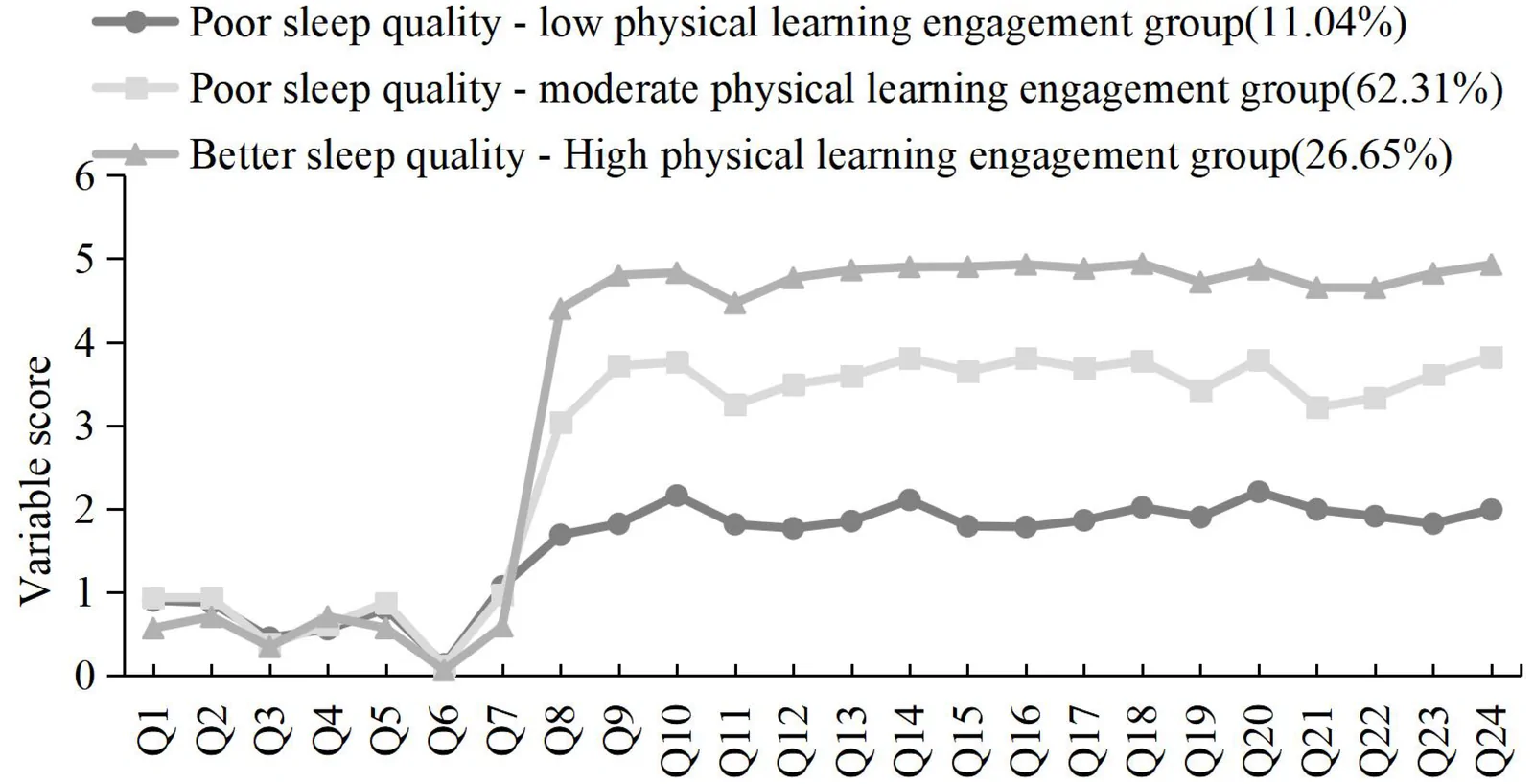
Distribution of characteristics across three potential categories of SQ and PLE.

Further comparisons of SA, SE, SA and PLE across the latent classes are presented in **Table 8**. Analysis of variance showed that the class differences were statistically significant for both SA and SE. These findings suggest that the latent classification based on SQ and PLE captured meaningful heterogeneity in students’ sleep- and learning-related patterns and was associated with significant differences in SA and SE. However, the latent profiles should not be interpreted as representing a simple ordinal hierarchy of overall psychological adjustment, because more favorable SQ–PLE configurations did not uniformly correspond to the most favorable levels of SE (see **Table 8**).

**Table 8:** Test of differences in SA, SE, SQ and PLE across different latent categories.

Variable	C1(125)	C2(702)	C3(303)	*F*	*p*	*Post hoc* comparisons
SA	37.17 ± 15.55	37.57 ± 14.09	31.70 ± 14.41	18.257	<0.001	C2>C1>C3
SE	25.36 ± 5.39	24.96 ± 5.30	22.90 ± 5.74	16.012	<0.001	C1>C2>C3
SQ	4.81 ± 3.24	4.83 ± 3.10	3.55 ± 3.12	18.525	<0.001	C2>C1>C3
PLE	32.51 ± 10.02	60.72 ± 6.78	81.28 ± 4.90	2294.404	<0.001	C3>C2>C1

Overall group differences were examined using one-way ANOVA. When the omnibus test was significant, Bonferroni-adjusted *post hoc* pairwise comparisons were conducted.

## 5 Discussion

### 5.1 Direct association between smartphone addiction and self-esteem among college students

The present study found a significant association between SA and SE among university students, and this relationship remained after controlling for SQ, PLE, gender, and age. This pattern suggests that the observed association between SA and SE was not fully accounted for by the indirect pathways examined in the current model and retained independent statistical significance within this cross-sectional dataset. Previous studies have generally suggested a robust relationship between problematic smartphone use and lower SE. For example, a large-scale study reported a significant negative correlation between problematic smartphone use and SE (67). Across university student samples from different cultural backgrounds, lower SE has likewise been identified as an important correlate or predictor of SA, with lower SE corresponding to greater levels of SA (68, 69). Taken together, the accumulated evidence generally supports the existence of a stable association between SA and SE. However, because the observed direction was positive rather than negative, this finding should not be interpreted as confirming dominant theoretical expectations; instead, it should be regarded as an unexpected and exploratory pattern requiring replication.

Contrary to mainstream theoretical expectations and numerous existing empirical studies, this study found a positive correlation between mobile phone addiction and self-esteem among college students. One possible explanation is that the psychological meaning of higher smartphone addiction scores may not be uniform across college students. In contemporary university contexts, smartphones function not only as potential sources of distraction and compulsive use but also as major tools for social interaction, self-presentation, relationship maintenance, and access to interpersonal feedback (70). As a result, higher levels of smartphone involvement may, for some students, coexist with more frequent online interaction, stronger perceived connectedness, and greater opportunities to receive social affirmation (71–73). Under such circumstances, elevated smartphone addiction scores may partly coincide with patterns of smartphone use that are socially rewarding rather than exclusively maladaptive (74). This possibility is particularly relevant in student populations, in which digital communication is deeply integrated into academic coordination, peer bonding, and everyday identity expression (75).

Another possibility is that qualitatively different patterns of smartphone use may have different implications for self-evaluation (76). Active and socially rewarding smartphone use, such as content sharing, communication with friends, and reciprocal online interaction, may be associated with belongingness, visibility, and positive self-presentation, whereas passive, comparison-oriented, or emotionally avoidant use may be more likely to undermine self-esteem (76–78). If these different forms of use are mixed within a global measure of smartphone addiction, the resulting association with self-esteem may not align with the dominant pattern reported in previous studies (79). In this sense, the present finding may reflect heterogeneity within problematic smartphone involvement.

At the same time, this interpretation remains inferential. The present study did not directly assess smartphone use motives, active versus passive use patterns, social comparison orientation, or the extent to which smartphone use was embedded in supportive versus stressful interpersonal contexts. Therefore, although the heterogeneity of smartphone use provides a plausible substantive explanation for the positive association observed here, it cannot be empirically verified on the basis of the current data. Future studies should distinguish more explicitly between different forms of smartphone engagement and test whether the association with self-esteem varies across usage motives, interaction styles, and social reinforcement patterns.

Overall, the present finding does not suggest that smartphone addiction is beneficial for self-esteem. Rather, it indicates that the association may be more complex and context-dependent than commonly assumed. Further studies should examine different forms of smartphone use and replicate this pattern in other samples.

### 5.2 The mediating role of sleep quality

The results further showed that SQ significantly mediated the relationship between SA and SE among university students. Compared with the other indirect pathways tested, this pathway appeared relatively more stable at the statistical level, suggesting that sleep-related factors may represent one potentially relevant correlate in understanding the association between SA and SE. Previous research has relatively consistently shown that problematic smartphone use is closely associated with poorer SQ. Demirci (80), for example, found in a university student sample that greater severity of smartphone use was associated with poorer SQ. A systematic review also indicated a relatively stable relationship between problematic smartphone use and sleep disturbance, particularly among adolescents and young adults (81). More recent work has further demonstrated that SA, as well as objectively measured screen time and unlocking frequency, are significantly associated with poorer SQ and shorter sleep duration (82). These findings are broadly consistent with the present result showing a significant positive association between SA and sleep problems. Importantly, because poorer sleep quality was positively rather than negatively associated with self-esteem in this sample, the indirect effect through sleep quality should be interpreted as a statistical pattern of uncertain substantive meaning rather than as robust support for an established theoretical mechanism.

However, in the present study, the statistical direction indicated that more severe sleep problems were associated with higher SE, a result that is inconsistent with most theoretical expectations and prior empirical findings. One possible explanation is that the relationship between subjective sleep quality and self-esteem may not be uniformly negative in all university student groups. For some students, poorer sleep quality may coexist with relatively high explicit self-evaluation when they are highly socially active, strongly achievement-oriented, or deeply involved in multiple academic and extracurricular demands (83, 84). In such cases, sleep complaints may reflect high levels of daily activation, intense role involvement, or heavy engagement in study and social life rather than generalized self-devaluation (29). From this perspective, poorer sleep quality may in some circumstances accompany lifestyles that also sustain a sense of competence, visibility, or personal worth, even if such lifestyles are not necessarily healthy.

A second possibility is that the present finding reflects the distinction between explicit self-esteem and more vulnerable or unstable forms of self-evaluation. The Rosenberg Self-Esteem Scale primarily assesses global explicit self-esteem, whereas poor sleep may be more closely related to emotional vulnerability, stress reactivity, or fluctuations in self-worth that are not fully captured by a brief global self-report measure (85–87). Some students may therefore report relatively positive explicit self-evaluations despite experiencing sleep problems, particularly when their self-esteem contains defensive or unstable components (85, 88). In addition, the PSQI measures subjective sleep quality rather than objective sleep parameters, and previous research has shown that subjective and objective sleep indicators do not always converge (89). Accordingly, the observed positive association may reflect a more complex interplay between self-perception, emotional functioning, and subjective symptom reporting.

Nevertheless, these interpretations should remain highly cautious. The present study did not include objective sleep indicators, perfectionism, achievement pressure, defensive self-esteem, or related variables that might help explain why poorer sleep quality was positively associated with self-esteem in this sample. Therefore, the current findings should not be interpreted as suggesting that sleep problems enhance self-esteem. Rather, they should be viewed as unexpected and exploratory, and the corresponding indirect effect through sleep quality should be understood primarily as a statistical pattern of uncertain substantive meaning that requires replication and more fine-grained theoretical examination.

Overall, although sleep quality emerged as the more consistently supported indirect pathway in the present study, the meaning of this pathway remains constrained by the anomalous direction of the association between sleep quality and self-esteem. Future research should combine subjective and objective sleep measures, differentiate stable versus vulnerable forms of self-esteem, and adopt longitudinal designs to determine whether this pattern reflects a reproducible subgroup-specific phenomenon or a sample-specific anomaly.

### 5.3 The mediating role of physical learning engagement

This study also examined the mediating role of PLE in the relationship between SA and SE, and this effect was primarily observed among male students. However, the overall effect was weak, and in the regression model, the association between PLE and SE did not reach statistical significance. Accordingly, this result should be interpreted cautiously and within limited scope. PLE may represent a potential behavioral pathway associated with SA and SE, although its stability, generalizability, and practical importance remain insufficiently supported.

Previous research provides some support for the first segment of this pathway. Kokoç (90) found that SA reduces university students’ engagement and disrupts the positive relationship between self-regulation and student engagement. Li (8) similarly reported that problematic smartphone use may affect school engagement and disengagement through academic procrastination and SQ. In the context of physical education, which relies heavily on bodily participation, sustained effort, and immediate feedback, the attentional distraction and reduced behavioral control associated with SA may theoretically be even more likely to undermine classroom engagement. Moreover, prior studies have indicated that physical activity is negatively associated with problematic smartphone use, whereas lower levels of activity are linked to greater burnout and poorer adaptation (91, 92). Together, these findings provide some support for the path from SA to PLE.

However, evidence for the second segment of the pathway, namely the relationship between PLE and SE, was relatively limited in the present study. Although greater engagement in physical education may promote positive self-evaluation by enhancing experiences of competence, physical self-identity, and the accumulation of positive feedback (93, 94), the corresponding path did not reach conventional significance in this sample (95). This suggests that the effect of PLE on SE may be weak in the present sample, or may be more strongly shaped by unmeasured factors such as prior exercise habits, classroom climate, teacher support, peer relationships, and evaluation methods. Therefore, the current findings are insufficient to regard PLE as a robust mediating mechanism. Overall, the index of moderated mediation did not reach significance, and thus gender differences should not be overinterpreted. At most, the present findings provide preliminary evidence that PLE may serve as a potential behavioral pathway. Future studies should adopt more refined indicators of physical education participation and further test the boundary conditions of this pathway across different course types, exercise experiences, and classroom support contexts.

### 5.4 The serial mediating role of sleep quality and physical learning engagement

The present study further examined the chain mediating role of SQ and PLE in the relationship between SA and SE. The results showed that this chained pathway was not statistically significant at the overall level, and only a weak trend was observed among male students, with limited overall robustness. Therefore, the current data do not provide sufficient support for a stable sequential association pattern linking SA, SQ, PLE, and SE.

Several reasons may account for this pattern. First, the relationship between SA and poorer SQ appears relatively stable (82, 96). Second, insufficient sleep recovery may lead to accumulated fatigue, reduced daytime alertness, and diminished behavioral initiative, thereby undermining students’ concentration, vitality, and sustained participation in physical education classes (97, 98). On this basis, if PLE is further associated with self-evaluation, the theoretical framework of “digital behavior–sleep recovery–educational engagement–self-evaluation” would be conceptually plausible. In other words, the chain mediation model proposed in this study remains theoretically plausible, but the present sample provided only limited empirical support for it.

Nevertheless, despite its theoretical plausibility, the bootstrap confidence interval for this chained pathway included zero, and it therefore cannot be interpreted as an empirically established mechanism. Several factors may explain this non-significant result. The actual association between SQ and PLE may be too weak (99). In addition, PLE may be more strongly influenced by external factors such as course context, teacher organization, and students’ prior exercise background (100, 101). Furthermore, SE is a broad psychological construct shaped by multiple influences (102), which may reduce the unique explanatory power of a complete chained pathway. Therefore, the present study is better understood as proposing this chained pathway as a theoretically feasible hypothesis rather than a robustly validated empirical conclusion.

In sum, SQ and PLE may jointly participate in the complex relationship between SA and SE, but the present study did not provide sufficient evidence for a stable sequential mediating effect. It should also be emphasized that statistical significance should not be equated with practical significance. Although some indirect effects reached statistical significance, their magnitudes were small, suggesting limited practical importance at the individual level. Given the relatively large sample size, these mediation findings are better viewed as preliminary evidence for possible mechanisms than as strong support for substantial applied implications. Future research should further test the stability and temporal ordering of this pathway by adopting longitudinal designs, refining measurement strategies, and examining whether these effects are replicable and practically meaningful across different contexts.

### 5.5 The moderating role of gender

The present study found that gender significantly moderated the direct association between SA and SE, whereas its moderating effects on the indirect pathways were not stably supported. This indicates that gender differences may be reflected primarily in the direct linkage between SA and self-evaluation, rather than consistently in mediating processes such as SQ or PLE. Therefore, when interpreting the relationship between SA and self-evaluation among university students, it is important to consider gender-related differences in smartphone use motivations, situational experiences, and psychological meanings (103). Previous studies have likewise suggested that male and female university students may differ in the manifestations and psychological consequences of problematic smartphone use (14, 104), and that gender may moderate the relationships between SA and variables such as negative affect and sleep disturbance.

In terms of possible mechanisms, female university students may use smartphones more frequently for social interaction, relationship maintenance, and feedback monitoring, making smartphone use more likely to become intertwined with social comparison, peer evaluation, and relational sensitivity, and thus more directly linked to self-evaluation (105). By contrast, male students’ smartphone use may more often be characterized by entertainment, instrumentality, or task orientation, and may therefore show a relatively weaker direct association with global SE (106). However, these explanations were not directly measured in the present study and thus remain speculative interpretations grounded in prior literature. At the same time, the observed gender differences should not be overstretched. Since the indices of moderated mediation were not significant except for the direct path, the effect of gender on the overall mechanism remains insufficiently stable. Gender may be an important source of heterogeneity in the relationship between SA and SE, but the specific psychological mechanisms through which it operates require further investigation. Future studies should incorporate variables such as smartphone use motivation, social comparison tendency, emotional sensitivity, and self-concept construction to provide a more fine-grained analysis of this heterogeneity.

### 5.6 Latent profile analysis

Beyond variable-centered analyses, the present study further employed latent profile analysis to examine the combined characteristics of sleep problems and PLE from the perspective of individual heterogeneity. The results indicated that university students could be classified into three distinct groups, and that these classes differed significantly in SA and SE levels (35, 107). This suggests that SQ and PLE may not be configured in the same way across all individuals; instead, students in different profile configurations may show distinct patterns of SA and SE.

It should be noted that the classes identified by latent profile analysis are essentially statistical groupings derived from sample data rather than sharply bounded and naturally existing categories (107, 108). Their main value lies in revealing heterogeneity within the population. Previous studies have likewise found latent classification features in smartphone use or SA among university students, with different classes showing significant differences in emotion, personality, and adaptation outcomes. The present findings are generally consistent with this broader trend.

At the same time, the profile characterized by lower sleep problems and higher PLE did not exhibit the highest level of SE. This pattern suggests that the latent profiles should not be interpreted as a simple continuum of psychological adjustment ranging from “less adaptive” to “more adaptive.” In other words, more favorable sleep and engagement characteristics do not necessarily correspond to uniformly higher SE. One possible explanation is that SE is shaped by a broader set of influences beyond sleep and PLE, including social comparison processes, perceived peer evaluation, emotional experiences, academic stress, and self-regulatory characteristics (14, 104). Students with relatively favorable behavioral functioning may still evaluate themselves less positively if they are exposed to stronger competitive pressures, more demanding achievement standards, or more frequent upward social comparison (109).

In addition, the profile with better sleep and higher engagement may not represent a uniformly advantaged group in all psychological respects. Greater learning engagement, although generally adaptive, may in some cases coexist with elevated achievement concerns, stronger performance expectations, or greater sensitivity to external evaluation (110). Under such conditions, the potential benefits of better sleep and higher engagement for self-esteem may be offset by pressure-related or comparison-based processes (109, 111). It is also important to recognize that each latent profile may still contain substantial within-profile heterogeneity (112). That is, individuals assigned to the same profile are similar only with respect to the observed indicators used in the model, but they may differ meaningfully in personality traits, coping style, interpersonal support, or academic demands (113).

Therefore, the value of latent profile analysis in the present study does not lie in constructing a simple “high-risk versus low-risk” ranking, but rather in identifying more complex combinations of sleep and engagement characteristics that relate differently to smartphone addiction and self-esteem. Future research should incorporate a broader range of psychological, interpersonal, and academic indicators to further clarify the adjustment characteristics and developmental implications of different profile groups.

## 6 Limitations and future directions

Despite the contributions of the present study, several limitations should be acknowledged. First, the study employed a cross-sectional design, and causal or temporal inferences should therefore be avoided or made only with great caution. In particular, the observed associations may also reflect reverse or reciprocal causality. Future studies should adopt longitudinal or experimental designs to further verify the temporal ordering and causal relations among the variables.

Second, all variables were measured using self-report questionnaires collected at a single time point, which makes the findings vulnerable to shared method variance. Although Harman’s single-factor test and a single-factor CFA were conducted as statistical checks for common method bias, these approaches provide only limited diagnostic evidence and do not fully eliminate the possibility of common method bias. Therefore, shared method variance may still have inflated or otherwise influenced the observed associations. Future studies should incorporate multi-source and multi-method data, such as smartphone usage logs, sleep monitoring, peer or teacher ratings, and behavioral observations, to reduce this concern and improve measurement accuracy.

Third, the explanatory power of the models was modest, and several observed effects were relatively small. Given the relatively large sample size, the analyses may have been sensitive to detecting very small indirect effects that reached statistical significance despite having limited practical magnitude. In addition, residual confounding from unmeasured variables cannot be ruled out. Factors such as depression, anxiety, family environment, personality traits, and academic burden may also have contributed to the observed associations. Therefore, the practical implications of the present findings should be viewed as preliminary rather than definitive.

Fourth, the sample was drawn from only two universities in Hunan Province, which limits representativeness. Future research should include broader geographic regions and different types of institutions to enhance the external validity of the findings.

Finally, the mechanisms linking SA and SE may also involve other variables, such as self-control, fear of missing out, social comparison, loneliness, and academic stress. Future work may build more comprehensive multiple-mechanism models on this basis.

## 7 Conclusion

The present study showed that SA was significantly associated with SE among university students. However, because the observed associations involving SE were directionally counterintuitive, they should be interpreted as unexpected and exploratory rather than as robust confirmation of prevailing theoretical expectations. Among the proposed indirect pathways, SQ emerged as the more consistently supported pathway, whereas the indirect effect through PLE was weaker and should be interpreted as preliminary and subgroup-specific, as it reached statistical significance only in the male subgroup. The hypothesized serial pathway through SQ and PLE was not supported. In addition, gender moderated the direct association between SA and SE, but there was no evidence of moderated mediation. LPA further identified three subgroups based on SQ and PLE that differed significantly in SA and SE. These findings should be interpreted cautiously in light of the cross-sectional design, the modest effect sizes, and the counterintuitive direction of some observed associations, and their practical implications should not be overstated.

## Data Availability

The original contributions presented in the study are included in the article/supplementary material. Further inquiries can be directed to the corresponding author.
