# Correction: Smartphone addiction and self-esteem among university students: a moderated mediation and latent profile analysis

**DOI:** 10.3389/fpsyt.2026.2009437

**Published:** 2026-10-05

**Authors:** Shiyu Zhang, Xiao Zhang, Siyu Yuan, Tianpeng Yang, Liang Li

**Affiliations:** 1 Physical Education College, Jiangxi Normal University, Nanchang, China; 2 Physical Education College, Shanghai University of Sport, Shanghai, China; 3 Physical Education College, Jiangxi Vocational and Technical College of Information Application, Nanchang, China; 4 Physical Education College, Nanchang Normal University, Nanchang, China; 5 Physical Education College, Jiujiang University, Jiujiang, China

**Keywords:** physical learning engagement, self-esteem, sleep quality, smartphone addiction, university students

There was a mistake in **Figure 2** as published. A Chinese label “性别 Int” was mistakenly retained within the path-diagram figure panel, which should read “Gender Int”. The corrected **Figure 2** appears below.

**Figure 2:**
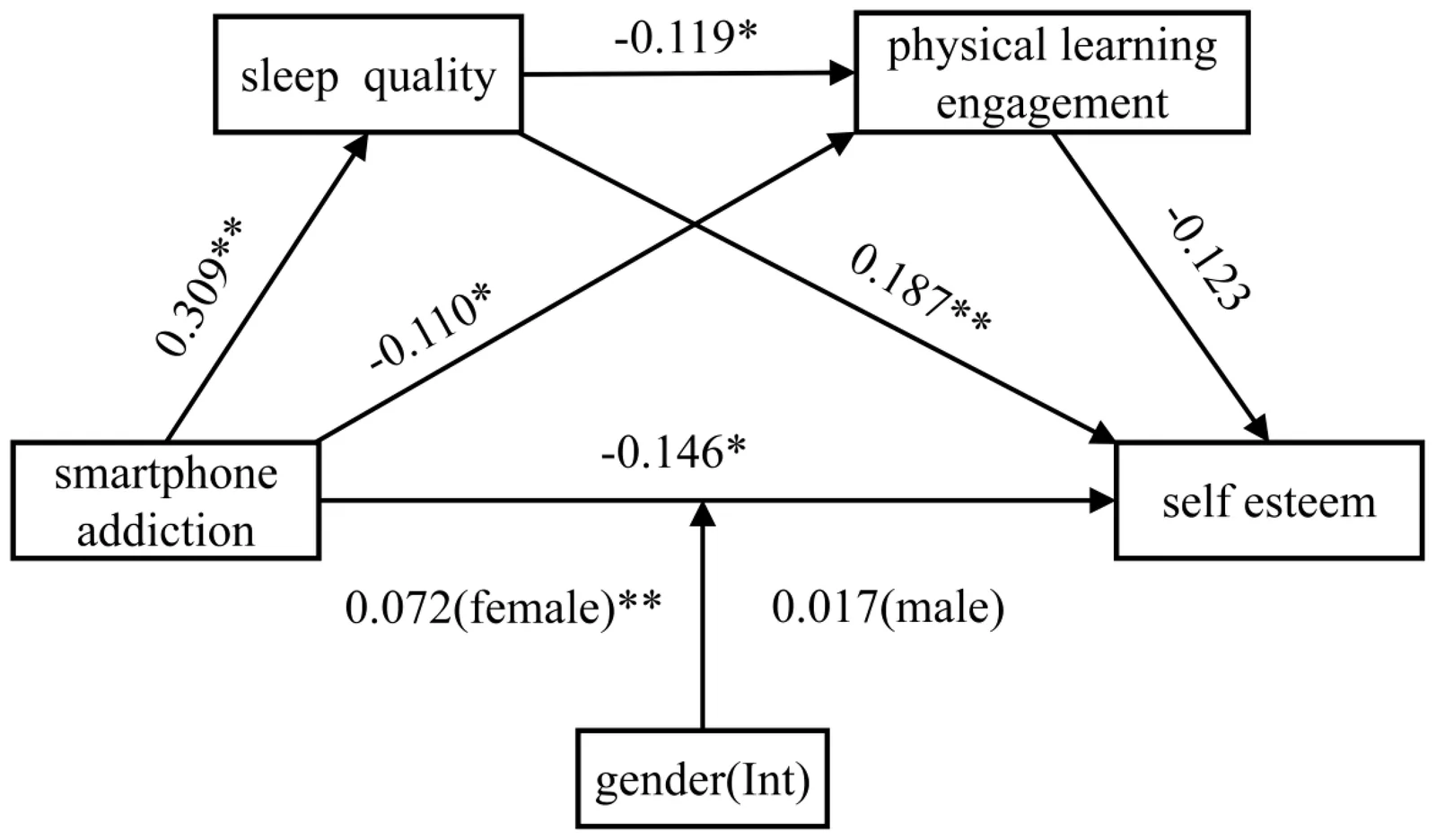
A moderated chain mediation effect model. Excluding paths with insignificant interaction effects (p>0.05). *p<0.05; **p<0,001.

The original version of this article has been updated.

